# Detection of *Mycobacterium tuberculosis* and *Mycobacterium bovis* in Sahiwal cattle from an organized farm using ante-mortem techniques

**DOI:** 10.14202/vetworld.2016.383-387

**Published:** 2016-04-15

**Authors:** Gursimran Filia, Geeta Devi Leishangthem, Vishal Mahajan, Amarjit Singh

**Affiliations:** Animal Disease Research Centre, Guru Angad Dev Veterinary and Animal Sciences University Ludhiana, Punjab, India

**Keywords:** bovine gamma interferon assay, bovine tuberculosis, *Mycobacterium bovis*, *Mycobacterium tuberculosis* complex, polymerase chain reaction, single intradermal comparative cervical tuberculin test

## Abstract

**Aim:**

The aim of this study was to investigate the prevalence of bovine tuberculosis (TB) and detection of Mycobacterium bovis in cattle from an organized dairy farm.

**Materials and Methods:**

A total of 121 animals (93 females and 28 males) of 1 year and above were studied for the prevalence of bovine TB using single intradermal comparative cervical tuberculin (SICCT) test, bovine gamma-interferon (γ-IFN) enzyme immunoassay, and polymerase chain reactions (PCRs).

**Results:**

Out of total 121 animals, 17 (14.04%) animals were positive reactors to SICCT test while only one (0.82%) animal for γ-IFN assay. By PCR, Mycobacterium TB complex was detected in 19 (15.70%) animals out of which 4 (3.30%) animal were also positive for M. bovis.

**Conclusions:**

Diagnosis of bovine TB can be done in early stage in live animals with multiple approaches like skin test followed by a molecular technique like PCR which showed promising results.

## Introduction

Bovine tuberculosis (TB) is an important infectious disease of cattle caused by *Mycobacterium bovis*, a member of the *Mycobacterium* TB complex (MTC). Other members of MTC include MTB, *M. bovis*, *Mycobacterium microti*, *Mycobacterium africanum*, *Mycobacterium canettii*, *Mycobacterium caprae*, *Mycobacterium suricattae*, etc. Besides cattle, it affects other domestic animals, wildlife, and also humans with worldwide annual losses to agriculture of $3 billion [1-3]. The disease is worldwide in distribution and is reported from many countries of the world including India. It is estimated that 300,000 people die from TB each year in India [4]. This zoonotic disease continues to have considerable economic and public health implications. Since both the species of *Mycobacterium* (MTB and *M. bovis*) pose a threat to health of animals and thereby capable of infecting humans and *viz*. (reverse zoonosis), detection of the bacteria in the early stage is needed.

Bovine TB in infected herd may occur due to the persistence of the microorganism in the environment [5] or because of its introduction in a previously free herd. Furthermore, indirect transmission due to the presence of infected goats in the farm could contribute to the recirculation of bovine TB within the cattle herd [6,7]. The purchase of infected animals and the interaction with infected cattle or goats at common pastures could be the external sources of bovine TB [8]. Intradermal tuberculin test is recognized by the World Organization for Animal Health (OIE) as the primary screening test for detection of bovine TB in cattle [9]. The application of this test, supplemented with molecular techniques like polymerase chain reaction (PCR) help in the detection of the disease in infected or suspected animals.

Thus, this study was conducted to investigate the prevalence of natural infection of bovine TB and detection of *M. bovis* in cattle from an organized dairy farm. The study would help in the differentiation of the animals suffering from the pathogenic mycobacteria and non-pathogenic mycobacterium. The animals suffering from pathogenic *M. bovis* can be segregated from the herd to prevent the spread of the disease.

## Materials and Methods

### Ethical approval

This study was approved by Animal Ethics Committee of Guru Angad Dev Veterinary and Animal Sciences University (GADVASU).

### Number of animals

The study was conducted in Bathinda district of Punjab. This organized farm did not have previous records on animals with confirmed *M. bovis* infection as the herd had not been previously skin tested. A total of 121 animals (93 females and 28 males) of 1 year and above were included in the study. 22 females were between 1 and 3 years of age, 39 were between 3 and 5 years of age and 32 were above 5 years of age. Out of 93 females, 81 were Sahiwal breed of cattle and 12 were crossbred.

### Single intradermal comparative cervical tuberculin (SICCT) test

This test compares immune responses to *M. bovis* (bovine) and *M. avium* (avian) tuberculin in the cervical region. All the animals were subjected to comparative cervical intradermal tuberculin test as per the guidelines from the World Organization for Animal Health (OIE). Briefly, the test was carried out in the middle third of the neck of each animal where avian tuberculin PPD-2500 (PPD-A) (Prionics) and bovine tuberculin PPD-3000 antigens (PPD-B) (Prionics) were injected (i.e., 0.1 ml of PPD) in two sites of neck 12 cm apart. Skin thicknesses were measured with caliper before and 72 h after PPD injections. The result is expressed as: (a) Positive reactor: Difference of the skin thickness at the injection sites is at least 4 mm greater, (b) negative reactor: No reaction to the bovine antigen or the difference of the skin thickness at the injection sites does not exceed 2 mm, while (c) inconclusive reaction: Reaction to both PPD-B and PPD-A exceeded 2 mm, but the difference between the bovine and avian reaction was <4 mm.

### Bovine gamma-interferon (γ-IFN) enzyme immunoassay (EIA)

Blood samples were collected from jugular veins in commercially available sterile 10 ml heparinized tubes a day before SICCT was conducted. The immunoassay was performed according to the manufacturer’s instructions. Briefly, in this test system, 1.5 ml aliquots of heparinized blood was dispensed into individual wells of 24-well tissue culture plates and incubated with 100 µl each of stimulating antigens (PPD-B and PPD-A) and PBS (non-stimulating control) for 16-24 h at 37°C in a humidified atmosphere with 5% CO_2_. The plasma was then collected and assayed for γ-IFN production in duplicate using acommercially available EIA kit (Bovigam^®^, Prionics, The Netherland), and optical densities were measured on an ELISA plate reader (Multiskan, MTX Lab Systems, Inc., USA) at 450 nm.

### Extraction and detection of DNA from blood samples

DNA was extracted from the blood samples by a modification of a QIAamp Blood and Tissue Kit (Qiagen). 1 ml of blood was taken and centrifuged at 14,000 rpm for 15 min. The cell pellet was suspended in lysis buffer, and further DNA extraction was done as per the manufacturer’s protocol. DNA was eluted and stored at −80°C. PCR reactions were performed as per Hermans *et al*. [10] and Rodriguez *et al*. [11] with slight modification in a reaction mixture (25 µl) containing 12.5 µl of Taq PCR master mix (Qiagen), 5 µl of DNA template and 0.2 µM of each primereg INS1 (forward)5’- CGTGAGGGCATCGAGGTGGC-3’, (INS 2) (reverse)5’-GCGTAGGCGTCGGTGACAAA-3’ for MTC and JB21 (forward) 5’-TCGTCCGCTGATGCAAGTGC-3’, JB22 (reverse) 5’-CGTCCGCTGACCTCAAGAAAG-3’ for *M. bovis*. Along with sample DNA, a known positive control DNA (Genekam Biotechnology AG, Germany) and a negative control was also amplified. Thermal cycling was performed in T Gradient Thermocycler (Biometra, Germany) with the following cycling parameter for INS1 and INS2: 94 for 5 min followed by 30 cycles of 1 min at 94°C, 1 min at 63°C and 1 min at 72°C followed by final extension of 7 min at 72°C. The cycling parameters for JB21 and JB22 were: 94 for 5 min followed by 35 cycles of 1 min at 94°C, 1 min at 68°C and 1 min at 72°C followed by final extension of 10 min at 72°C and PCR products were run by agarose gel electrophoresis using 1.5% agarose gel and visualized in gel documentation system (Bio-Rad, CA).

## Results

### SICCT test and γ-IFN assay

Of 121 animals tested by SICCT test, 17 (14.04%, 95% confidence intervals [CI] = 8.14-20.66) animals were positive reactors, 27 (22.31%, 95% CI = 14.88-29.72) were inconclusive while 77 (63.63%, 95% CI = 55.06-72.2) were negative reactors. Among 17 animals tested positive, 14 were Sahiwal females and 3 crossbreed females. All males were negative. The prevalence of positive reactors in Sahiwal breed was 17.28% (14 out of 81) and in crossbred it was 25% (3 out of 12). Only one (0.82%, 95% CI = 0.79-2.43) crossbreed animal was tested positive to γ-IFN assay.

### Detection of MTC and M. bovis by PCR

**Figure-1:**
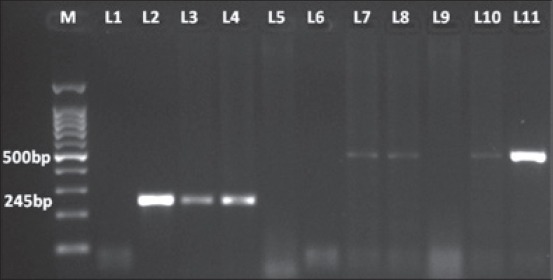
Amplification of DNA from the samples using INS1/INS2 primers and JB21/JB22 (M: 100 bp plus molecular weight marker, L1: Negative control, L2: Positive control for *Mycobacterium tuberculosis* complex (MTC), L11: Positive control for *Mycobacterium bovis*, L3 and L4: Samples positive for MTC, L7, L8 and L10: Samples positive for *M. bovis*, L5, L6 and L9: Samples negative).

PCR using primers INS1/INS2 was successfully amplified at target regions of 245 bps fragment diagnostic for MTBC in 19 (15.7%, 95% CI = 9.22-22.18) animals (16 Sahiwal, 3 crossbred) which include 17 positive reactor and 2 inconclusive reactors by SICTT test. PCR using primers JB21/JB22 has amplified target regions of 500 bps fragment specific for *M. bovis* in 4 (3.3%, 95% CI = 0.12-6.48) Sahiwal breed of animals (Table-1). Agarose gel electrophoresis of the products of genomic DNA from samples using primers specific for MTC and *M. bovis* are shown in Figure-1. The PCR result of the only positive γ-IFN sample was also positive for MTC.

**Table-1:** Results of SICCT, γ-IFN assay and PCR.

Breed	SICCT results (%)	γIFN assay	PCR for MTC (%)	PCR for *M. bovis* (%)
Sahiwal breed female (n=81)				
Positive	14 (17.28)	-	16 (19.75)	4 (4.90)
Inconclusive	23 (28.39)	-		
Negative	44 (54.32)	-	-	-
Crossbred female (n=12)				
Positive	3 (0.25)	1	3 (25)	-
Inconclusive	3 (0.25)	-		
Negative	6 (0.50)	-	-	-
Male animal (n=28)				
Positive	-	-	-	-
Inconclusive	1 (3. 57)	-	-	-
Negative	27 (96.42)	-	-	-
Total animal (n=121)				
Total positive	17 (14.04)	1 (0.82)	19 (15.70)	4 (3.30)
Total inconclusive	27 (22.31)	-		
Total negative	77 (63.63)	120 (99.17)		

*M. bovis: Mycobacterium bovis*, SICCT=Single intra-dermal comparative cervical tuberculin, IFN=Interferon, PCR=Polymerase chain reaction, MTC=*Mycobacterium tuberculosis complex*

## Discussion

Diagnosis of bovine TB in live animals is primarily based on the detection of specific cell-mediated immune (CMI) responses (the skin test and γ-IFN assay) [12,13] which occur as early as 3 weeks post *Mycobacterium* infection in cattle [14]. Although culture is considered to be the “gold standard” for confirming TB, this procedure is slow and takes several weeks.

In this study, skin test and γ-IFN test was conducted as a routine bovine TB screening procedure in an organized dairy farm. The previous data about *M. avium* subsp. Para-TB or other infection with non-TB *Mycobacterium* species in the region was not taken into consideration. It was found that by SICCT test out of 121 animals, 17 (14.04%) animals were positive reactors, 27 (22.31%) were inconclusive while 77 (63.63%) were negative reactors. However, γ-IFN assay showed only one (0.82%) positive animal. Similar to this result, few reports are available regarding a small proportion of *M. bovis*-infected cattle that are reactors to the skin tests are not detected by the γ-IFN test [15-17]. Breed wise higher prevalence of bovine TB among the crossbred animals than the Sahiwal cattle could be due to high production potential of crossbred animals and birth to a considerable number of young one, which is directly related to degree of stress on animals. Hence, the animal may succumb to active disease given such stress factors. Further by PCR, this animal was found positive for MTC but not for *M. bovis*. This may be due to the fact that the immune response to bovine TB is multifaceted and that diagnostic parameters are likely to perform differentially as disease progresses. Various factors such as age, animal genotype, the presence of intercurrent infections and possibly the virulence of the particular strain of *M. bovis* influenced the immune response, and therefore, the performance of CMI-based diagnostic tests [18]. Further, this assay was found to be particularly useful in detecting cattle during the initial stages of cellular responses, which dominate early infection by *M. bovis* [18].

For the rapid and specific diagnosis of TB, PCR assays are the most promising alternative method [19,20]. PCR techniques offer high sensitivity and have been successfully used for diagnosing bovine TB in several types of naturally infected organic materials such as tissue, blood, and nasal exudates [21,22]. The most commonly used system is based on primers that amplify segments of the IS*6110* element, particularly targeting 245 bps fragments. In this study, IS*6110* PCR was employed using specific primers (INS1/INS2) to amplify an insertion sequence IS*6110* of 245 bps in the MTC directly on DNA extracted from blood samples. The IS*6110* PCR assay was positive in 19/121 (15.70%) animals which include 17 positive reactor and 2 inconclusive reactors by SICTT test. This indicates that the samples were positive for MTC. There was a concordance of 14.04% between PCR for MTC and SICCT test.

Further, to detect and distinguish *M. bovis* from other members of MTC, all the samples were amplified using specific primers JB21/JB22 which target 500 bps DNA fragment inside the RvD1Rv2031c genomic sequence. *M. bovis* was detected in 4/121 (3.30%) animals which were also positive for MTC. PCR assays using primers JB21/JB22 have been considered to be highly reliable in identifying *M. bovis* isolates, showing 100% concordance with the conventional microbiological method [23]. Shah *et al*. [24] used a multiplex-PCR that allowed detection of a single product of 500 bp in *M. bovis* while MTB generated a single product of 185 bp, with or without an additional product of 500 bp. Lower detection of *M. bovis* has been reported by Sechi *et al*. [25], who reported that 13.3% (4/30) of *M. bovis* isolates failed to produce the 500-bps fragment. By using the primers internalized in the insertion sequence IS*6110*, Sechi *et al*. [25] confirmed that the isolates that remained unidentified by JB21/JB22 belonged to the MTC.

Thus, in this study both *M. bovis* and MTC were the aetiological agent for bovine TB. Many researchers stated that MTB (human TB) and *M. caprae* (goat TB) may also cause bovine TB. Further studies are required to identify the other member of MTC which is causing the disease in cattle.

## Conclusions

The presence of *M. bovis* and other potential pathogenic mycobacteria in animal may raise concerns regarding the zoonotic risk for humans, especially those living at the animal-human interface. Diagnosis of bovine TB can be done in early stage in live animals with a multiple approaches like skin test followed by molecular technique like PCR which showed promising results. This can lead to quick segregation of infected animals; restrict transmission and rapid eradication of bovine TB in the country. Large-scale studies are required for detection, control and eradication of bovine TB in the state.

## Authors’ Contributions

GF conceptualized the aim of the study, designed, planned, and supervised the experiments and corrected the manuscript. GF, VM and GDL performed the SICCT test, γ-IFN assay and molecular biology work. GDL and GF drafted the manuscript. AS provided conceptual support, and critically reviewed the manuscript. All authors read and approved the final manuscript.
